# Diagnostic Ability of Magnifying Narrow-Band Imaging for the Extent of Early Gastric Cancer: A Systematic Review and Meta-Analysis

**DOI:** 10.1155/2021/5543556

**Published:** 2021-04-23

**Authors:** Yingying Hu, Xueqin Chen, Maher Hendi, Jianmin Si, Shujie Chen, Yanyong Deng

**Affiliations:** ^1^Department of Gastroenterology, Sir Run Run Shaw Hospital, School of Medicine, Zhejiang University, Hangzhou, Zhejiang Province, China; ^2^Institution of Gastroenterology, Zhejiang University, Hangzhou, Zhejiang Province, China; ^3^Department of General Surgery, Sir Run Run Shaw Hospital, School of Medicine, Zhejiang University, Hangzhou, Zhejiang Province, China

## Abstract

**Background:**

Accurate delineation of tumor margin is essential for complete resection of early gastric cancer (EGC). The objective of this study is to assess the performance of magnifying endoscopy with narrow-band imaging (ME-NBI) for the accurate demarcation of EGC margins.

**Methods:**

We searched PubMed, EMBASE, Web of Science, and Cochrane Library databases up to March 2020 to identify eligible studies. The diagnostic accuracy of ME-NBI for EGC margins was calculated, and subgroup analyses were performed based on tumor size, depth of tumor invasion, tumor-occupied site, macroscopic type, histological type, *Helicobacter pylori* (*H. pylori*), and endoscopists' experience. Besides, we also evaluated the negative and positive resection rates of the horizontal margin (HM) of EGC after endoscopic submucosal dissection (ESD) and surgery.

**Results:**

Ten studies comprising 1018 lesions were eligible in the databases. The diagnostic accuracy of ME-NBI for the demarcation of EGC margins was 92.4% (95% confidence interval (CI): 86.7%-96.8%). According to ME-NBI subgroup analyses, the rate of accurate evaluation of EGC margins was not associated with *H. pylori* infection status, tumor size, depth of tumor invasion, tumor-occupied site, macroscopic type, histological type, and endoscopists' experience, and no statistical differences were found in subgroup analyses. Moreover, the negative and positive resection rates of HM after ESD and surgery were 97.4% (95% CI: 92.1%-100%) and 2.6% (95% CI: 0.02%-7.9%), respectively.

**Conclusions:**

ME-NBI enables a reliable delineation of the extent of EGC.

## 1. Introduction

Early gastric cancer (EGC) is identified as gastric cancer in which its invasion is confined to the mucosal or submucosal layer, regardless of lymph node metastasis [1]. Endoscopic submucosal dissection (ESD) has been widely used in the treatment of EGC, especially following the development of the expanded indications for ESD in the Japanese Gastric Cancer Association Guideline [2]. Not only differentiated-type early gastric cancer (D-type EGC) but also undifferentiated-type early gastric cancer (UD-type EGC) might be completely resected by ESD as long as it is an intramucosal lesion measuring ≤20 mm without ulceration or metastasis [2]. However, inaccurate delineation of tumor margins may induce incomplete resection with positive margins [3, 4]. Therefore, accurately demarcating the extent of EGC is essential and urgent.

In Japan, indigo carmine chromoendoscopy following conventional white-light imaging is regarded as a standard method to delineate the extent of gastric cancers [5]. Chromoendoscopy is widely used for endoscopic evaluation in EGC, but Nagahama et al. found it failed to demarcate the lateral margin in 18.9% of patients [6]. Recently, magnifying endoscopy with narrow-band imaging (ME-NBI) is reported to visualize the microvascular (MV) and microsurface (MS) patterns of gastric mucosa [7, 8]. Yao et al. [7, 8] have proposed a system called the “VS (vessel plus surface) classification system,” which enables the distinction of the cancerous lesions from noncancerous lesions. Furthermore, ME-NBI was also deemed useful in delineating the horizontal margin of EGC [6, 7], although its diagnostic accuracy was variable from 69% to 100% [9–18]. The capacity for demarcating the margins of EGC correctly by ME-NBI might be influenced by *Helicobacter pylori* (*H. pylori*) statues [12, 16], tumor-occupied site [17], and tumor size [17], among other factors. Therefore, the primary purpose of this meta-analysis was to evaluate the diagnostic accuracy of ME-NBI for the demarcation of EGC margins. As for ME-NBI, we also assessed the possible factors accounting for the accuracy in order to guide our endoscopic work accurately and convincingly.

## 2. Methods

### 2.1. Search Strategy

We searched the PubMed, EMBASE, Web of Science, and Cochrane Library databases for studies focusing on the delineation accuracy of ME-NBI for EGC margins in English up to March 2020. Additional manual searches from the reference lists of relevant studies were also conducted to identify eligible studies. The search terms were “gastric cancer,” “gastric carcinoma,” “gastric neoplasm,” “stomach cancer,” “stomach carcinoma,” “stomach neoplasm,” “narrow band imaging,” “NBI,” “demarcation,” “extent,” “margin,” and “DL.” This protocol was reported according to the PRISMA statement.

### 2.2. Inclusion and Exclusion Criteria

The inclusion criteria were as follows: (1) the goal of the articles was to evaluate the demarcation accuracy of the margins of EGC by ME-NBI; (2) the diagnostic accuracy of delineation of EGC margins could be obtained directly or calculated indirectly; (3) the diagnostic gold standard was histopathology and according to the revised Vienna classification, it was identified as Category 4 (mucosal high-grade neoplasia) or Category 5 (submucosal invasion of neoplasia) [19]; and (4) they were published as full articles in English.

The exclusion criteria were as follows: (1) the histopathology result was not the gold standard; (2) combined ME-NBI with other examinations, such as chromoendoscopy, to evaluate the diagnostic accuracy of EGC margins; (3) only contained lesions which could not be identified by white-light imaging or chromoendoscopy, followed by ME-NBI to evaluate; and (4) case reports, review articles, editorials, comments, meeting abstracts, and articles which only had abstracts.

### 2.3. Selection of Studies and Data Extraction

The studies were screened and assessed by two independent reviewers for inclusion. After scanning the titles and abstracts of articles, we reviewed the full text of potentially relevant studies. If discrepancies occurred, a third investigator would resolve the difference via discussion. We obtained the following information from each study: the first author, years, age, gender, number of patients, number of lesions, the criteria of endoscopic diagnosis, tumor size, depth of tumor invasion, tumor-occupied site, *H. pylori* status, macroscopic types, histological type, and endoscopists' experience. The positive resection number of the horizontal margins after ESD and surgery was also extracted.

### 2.4. Data Analysis

All statistical analyses were performed using R version 3.6.0 (meta package version 4.9-5). This meta-analysis was conducted to evaluate the delineation accuracy of the extent of EGC by ME-NBI. Heterogeneity among the included studies was assessed using the *I*^2^ statistic. When the *I*^2^ value was equal to or less than 50%, a fixed-effect model (Mantel-Haenszel method) would be chosen; otherwise, a random-effect model (DerSimonian-Laird method) was used. Results were assessed with 95 percent confidence intervals (95% CI), and it would be considered to be statistically significant if *P* value was less than 0.05. When heterogeneity was present, we conducted subgroup analyses to find the possible heterogeneity, according to tumor size, invasion depth, tumor-occupied site, macroscopic type, *H. pylori* infection status, histological type, and endoscopists' experience. Additionally, sensitivity analysis was applied to assess the stability of the article results. Publication bias was analyzed based on the funnel plot, as well as Egger's regression test.

### 2.5. Quality Evaluation

The quality of the included articles was evaluated using the Quality Assessment of Diagnostic Accuracy Studies (QUADAS), as shown in Supplementary Materials (Supplementary data 1). A total of 14 items were assessed, with each assessment estimated as “yes,” “no,” or “unclear.” The evaluation was assessed by two independent investigators, and disagreements were settled via discussion.

## 3. Results

### 3.1. Literature Search

**Figure 1:**
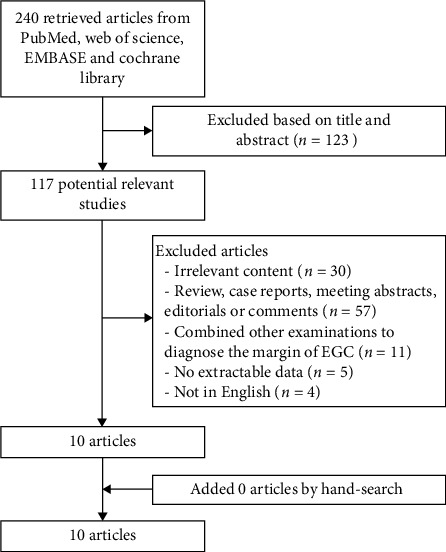
Flow diagram of study selection.

The systematic search yielded 240 potentially eligible studies from the PubMed, EMBASE, Web of Science, and Cochrane Library databases. After initial screening of titles and abstracts, 123 studies were excluded, leaving 117 articles for further analysis. Based on the selection process and exclusion criteria as showed in Figure 1, ten articles were included by electronic search [9–18] comprising 1018 lesions for final analysis.

### 3.2. Study Characteristics and Quality Assessment

The characteristics of the ten articles are presented in Table 1. They were all performed in Japan and evaluated the diagnostic value of ME-NBI on the demarcation of EGC margins. Besides, there were six studies involving the numbers of positive resection and negative resection of horizontal margins (HM) after ESD or surgery. Among them, five articles evaluated HM in UD-type EGC and two articles involved HM in D-type EGC. The details of overall quality of the selected studies are shown in Supplementary Materials (Supplementary data 2), according to the QUADAS questionnaires.

### 3.3. Diagnostic Performance of ME-NBI for the Extent of EGC

**Figure 2:**
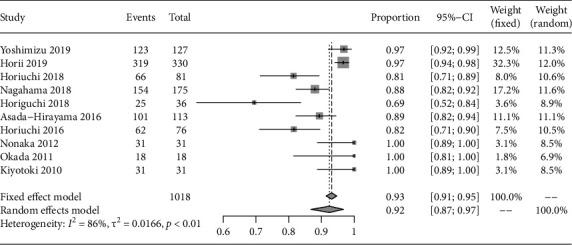
The diagnostic accuracy of magnifying endoscopy with narrow-band imaging for the extent of early gastric cancer.

According to the included articles, the diagnostic accuracy of the extent of EGC by ME-NBI was yielded as 92.4% (95% confidence interval: 86.7-96.8) (Figure 2), displaying an excellent performance in delineating margins of EGC. On account of significant heterogeneity between articles (*I*^2^ = 86.1%, *P* < 0.01), a random-effect model was performed.

### 3.4. Subgroup Analysis

As showed in Figure 2, there was large heterogeneity in the eligible articles. Due to exploring the potential sources of the heterogeneity, subgroup analyses were also performed, as showed in Table 2, through stratifying the data based on tumor size (≤20 mm vs. >20 mm), tumor-occupied site (upper, middle vs. lower third), macroscopic type (elevated, flat vs. depressed), invasion depth (T1a vs. T1b), *H. pylori* infection status (uninfected, eradication vs. noneradication), histological type (differentiated vs. undifferentiated), and endoscopists' experience (experienced (endoscopy experience of ≥5 years) vs. less experienced (endoscopy experience of <5 years)).

According to the results of subgroup analyses, we found that the rates of accurate delineation in the *H. pylori*-uninfected group, the *H. pylori* eradication group, and the non-*H. pylori* eradication group were 96.8% (95% CI: 75.3%-100%), 90.4% (95% CI: 74.0%-99.5%), and 85.5% (95% CI: 70.3%-96.1%), respectively, and there was no statistical significance (*P* = 0.82). As for the differentiated or undifferentiated type of EGC, demarcation accuracy was 92.8% (95% CI: 84.9%-98.1%) and 91.8% (95% CI: 82.3%-98.2%), respectively, where there was little difference between them (*P* = 0.68). In addition, tumor-occupied site, tumor size, macroscopic type, invasion depth, and endoscopists' experience were not significantly associated with accurate delineation of EGC margins at all (Table 2).

### 3.5. Horizontal Margin

**Figure 3:**
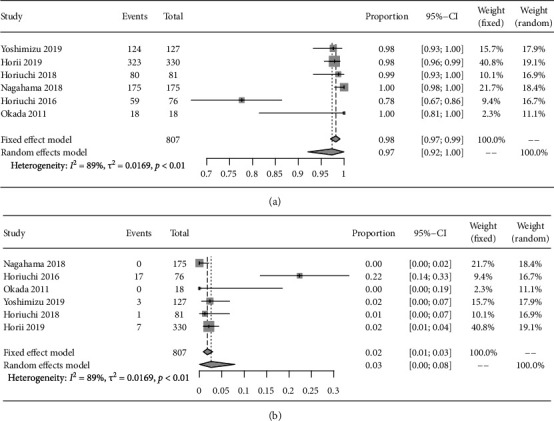
The negative and positive resection rates of HM of EGC after ESD and surgery. (a) The negative resection rates of HM of EGC after ESD and surgery. (b) The positive resection rates of HM of EGC after ESD and surgery. HM: horizontal margin; EGC: early gastric cancer; ESD: endoscopic submucosal dissection.

As for ME-NBI, six articles also evaluated the negative and positive resection rates of the horizontal margin (HM) after ESD and surgery, which were 97.4% (95% CI: 92.1%-1.0%) and 2.6% (95% CI: 0.02%-7.9%), respectively (Figure 3). What is more, the negative resection rate of HM in UD-type EGC and D-type EGC was 97.4% (95% CI: 89.1%-100%) and 99.8% (95% CI: 98.2%-100%), where significant difference was absent (*P* > 0.05) (Table 3).

### 3.6. Publication Bias

**Figure 4:**
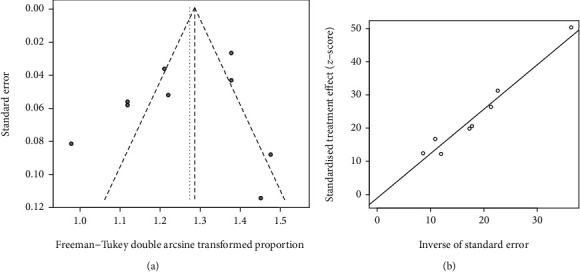
The funnel plot and Egger's regression test for publication bias. (a) The funnel plot for publication bias. (b) Egger's regression test for publication bias.

The publication bias of ten articles was assessed by the funnel plot and Egger's regression test (Figure 4). No obvious asymmetry was discovered in these studies, and the result of Egger's regression test also displayed no evidence of publication bias (*P* = 0.632).

### 3.7. Sensitivity Analysis

We applied a “leave-one-out” sensitivity analysis to identify the possible causes. No substantial variations were found after eliminating each study in turn, as shown in Supplementary Materials (Supplementary data 3).

## 4. Discussion

Nowadays, ME-NBI has gradually become popular in our endoscopic work [6]. Although ME-NBI was reported useful in the demarcation of the extents of EGC, the rate of diagnostic accuracy was inconsistent. To our knowledge, no meta-analysis has been performed on the delineation accuracy of EGC margins by ME-NBI. In response, we made this meta-analysis to assess the delineation accuracy of ME-NBI for EGC margins. The results of this study demonstrated that ME-NBI is a highly specific diagnostic tool for delineating the extent of EGC, with a rate of 92.4% (95% CI: 86.7%-96.8%). Moreover, we hypothesized that the diagnostic capacity of ME-NBI might be influenced by some possible causes, whereas we failed to identify the possible reasons which might be associated with the diagnostic performance of ME-NBI.

Chromoendoscopy is regarded as effective in the preoperative evaluation for the extent of early gastric cancer due to its ability to identify subtle changes in gastric mucosal epithelium involved with the horizontal spread of gastric cancer. However, several studies reported that the performance of chromoendoscopy was lower than that of ME-NBI associated with the demarcation of EGC margins [6]. As Nagahama et al. reported, chromoendoscopy could not identify 18.9% (66/350) of the margins of early gastric cancer lesions; however, 72.6% (45/62) of which could be successfully delineated by ME-NBI following chromoendoscopy [6]. Another retrospective, a single-center trial for early gastric cancers, showed a significant added benefit of ME-NBI, especially with the highest power optical magnifying endoscopy [20]. These studies predicted that ME-NBI could be reliable for the delineation of the extent of EGC and have a possible superiority over chromoendoscopy in the demarcation accuracy of EGC margins, which need further researches.

In our understanding, the “VS (vessel plus surface) classification system,” an irregular MV pattern and/or irregular MS pattern with the demarcation line, is mostly applied to identify early gastric cancer by ME-NBI. However, as reported, it is still difficult to assess the horizontal extent of UD-type early gastric cancer, possibly because of the lateral infiltration of cancer cell within the proliferative zone (PZ) [11, 21]. In our study, we found that the accuracy of delineation of UD-type EGC margins by ME-NBI was approximately 91.8% (95% CI: 82.3%-98.2%), which was almost similar to that of D-type EGC margins. It indicated that ME-NBI is not only beneficial for identifying the extent of D-type EGC but also for UD-type EGC margins. The infection of *H. pylori* is known to be closely correlated with gastric cancer, and several studies have reported that *H. pylori* eradication could reduce the risk of gastric cancer among patients with peptic ulcer disease [22]. However, with the increase of *H. pylori* eradication, it was still controversial whether the eradication of *H. pylori* was a benefit for the delineation of EGC. It was reported that after successful eradication of *H. pylori*, 4% of EGC showed a “gastritis-like” appearance by ME-NBI, resembling adjacent noncancerous mucosa, which might mislead us when identifying EGC margins [23]. Nevertheless, as Horiuchi et al. reported, *H. pylori* eradication leads to the reduction of neutrophil infiltration and a higher mean intercrypt distance ratio in the eradication group of UD-type EGC. Ultimately, *H. pylori* eradication promoted the accurate demarcation of UD-type EGC margins [24], whereas our study displayed the demarcation of EGC margins by ME-NBI was not obviously affected by *H. pylori* infection status.

According to the reports, the negative resection rate of horizontal margin in ESD for differentiated-type EGC was 96.9% to 99% [25–27], whereas the value for undifferentiated-type EGC was 72.7% to 94.8% [28–32]. In this study, the negative resection rate of horizontal margin after ESD and surgery was 97.4% (95% CI: 92.1%-1.0%) though there were almost no difference between that of UD-type EGC and D-type EGC. The negative resection rate of HM was found to be higher than the delineation accuracy of EGC margins, which may be due to the second biopsy after the first positive biopsy [17], and the extended resection is usually 5 mm outside the margin [33].

The current meta-analysis has several limitations. First, there was significant heterogeneity among the articles included, but the source of heterogeneity was not found through subgroup analysis, which might be caused by other factors influencing the heterogeneity that were not analyzed. Moreover, the factors of subgroup analyses were referred to in some articles, not in every eligible article. Second, the *H. pylori*-uninfected group contained far fewer lesions than the other groups, which only included 61 lesions in three articles. Although the prevalence of *H. pylori*-uninfected was reportedly about 1% in all gastric cancer patients [34, 35], more articles about EGC uninfected with *H. pylori* should be performed to analyze the accuracy further. Third, the biopsy specimens were taken from different locations because of the lack of uniform diagnostic standards. In the future, we could define unified diagnostic standards in order to analyze endoscopic diagnostic performance accurately. Fourth, the included studies were all from Japan, which might be due to the prevalence of early gastric cancer in Japan and their developed endoscopic technology.

## 5. Conclusion

In summary, this meta-analysis revealed that ME-NBI is an effective tool for the accurate delineation of the extent of early gastric cancer. Since there was large heterogeneity among the included articles, it may be necessary to investigate the diagnostic performance of ME-NBI for the margins of early gastric cancer further and establish a normalized diagnostic standard.

## Figures and Tables

**Table 1:** Characteristics of the ten included articles.

Study	Country	Study type	No. of patients	Sex, male/female	No. of lesions	Age, mean ± SD or range (years)	Diagnostic standard	Macroscopic type (elevated/flat/depressed)	Tumor size, mean ± SD or range (mm)	Location (upper third/middle third/lower third)	Histological type (differentiated/undifferentiated)	Depth of invasion (T1a/T1b and deeper)	*H. pylori* status (noneradication/uninfected/eradication/unknown)	Endoscopists' experience (experienced^a^/less experienced^b^)	No. of positive resection of horizontal margin
Yoshimizu et al. [14]	Japan	Retrospective	127	74/53	127	56 (26-80)	Four or more circumferential biopsies approximately 5 mm apart from the estimated lesion border were obtained to confirm noncancerous areas	7/26/94	12 (1-29)	1/86/40	0/127	119/8	NA	NA	3
Horii et al. [17]	Japan	Retrospective	330	251/79	330	NA	At least 4 biopsies were taken from noncancerous tissues approximately 5 mm outside the EGC lesion	146/184 (flat+depressed)	<20 (268 people) >20 (62 people)	58/272 (middle+lower)	NA	NA	212/2/116/0	330/0	7
Horiuchi et al. [16]	Japan	Retrospective	81	46/35	81	NA	Both the utmost oral and utmost anal sites marked in ME-NBI were consistent with the results of postoperative pathological examination	0/13/68	≤20	6/35/40	0/81	72/9	33/21/27/0	81/0	1
Nagahama et al. [15]	Japan	Prospective	175	118/57	175	69 ± 9	Biopsies were taken from noncancerous and cancerous mucosa, each at 5 mm from the margin on the oral-most side	36/9/130	24 ± 15	35/86/54	142/33	138/37	96/NA/NA/2	144/31	0
Horiguchi et al. [9]	Japan	Prospective	30	23/7	36	67.5 (57–83)	A complete match of the histological and endoscopic horizontal extents at all four circumferential quadrant points (oral, anal, anterior, and posterior sites) around the cancerous lesion	7/0/29	10.0 ± 4.4 (mean ± SE)	4/13/19	34/2	30/6	0/0/36/0	NA	NA
Asada-Hirayama et al. [12]	Japan	Prospective	103	73/30	109	73.3 ± 9.0 (accurate evaluation)/72.8 ± 7.5 (inaccurate evaluation)	The marking dots (oral or anal edge of the tumor) were located within 1 mm of the pathological tumor border	44/69	21.5 ± 13.7 (accurate evaluation)/31.0 ± 17.7 (inaccurate evaluation)	18/55/36	105/4	84/25	42/30/24/7	NA	NA
Horiuchi et al. [13]	Japan	Prospective	76	45/31	76	54.5 ± 11.3 (accurately diagnosed)/58.5 ± 10.0 (misdiagnosed)	Both the utmost oral and anal sites were consistent with the postoperative pathological examination	NA	9.95 ± 6.36 (accurately diagnosed)/19.07 ± 2.75 (misdiagnosed)	6/32/38	0/76	67/9	NA	76/0	17
Nonaka et al. [10]	Japan	NA	31	25/6	31	71 (57-87)	Biopsies were taken from noncancerous and cancerous mucosa, each at 1.8 mm from the margin on the orifice and anal sides of each lesion	0/7/24	22 (3-72)	8/15/8	31/0	NA	NA	NA	NA
Okada et al. [11]	Japan	Prospective	18	12/6	18	57.9 ± 10.4	The distance between an APC representing the oral and/or anal borders of the tumor and the pathological lateral extent of cancer was within 1 mm	0/11/7	8.1 ± 5.7 (1–20)	4/9/5	0/18	16/2	NA	NA	0
Kiyotoki et al. [18]	Japan	NA	NA	NA	31	NA	The distance between the marking dots (one or two marking dots on the tumor margin) and the tumor margin was less than 1 mm	NA	NA	NA	NA	NA	NA	NA	NA

T1a: mucosal cancer; T1b: submucosal cancer; EGC: early gastric cancer; ME-NBI: magnifying endoscopy with narrow-band imaging; APC: argon plasma coagulator; NA: not available. ^a^Experienced means >5 years of endoscopy experience. ^b^Less experienced means <5 years of endoscopy experience.

**Table 2:** Subgroup analysis on diagnostic accuracy of magnifying endoscopy with narrow-band imaging for the extent of early gastric cancer.

Study characteristics	Number of studies	*n* (lesions examined)	Events (lesions examined)	*I* ^2^	*P*	95% CI (%)	*P* value
Overall	10	1018	930	86.1%	<0.01	92.44 [86.74-96.75]	
Tumor size (mm)							
≤20	5	526	481	92.7%	<0.01	91.22 [78.40-98.91]	0.5728
>20	1	62	54	NA	NA	87.10 [77.45-94.46]	
Depth							
T1a	4	305	270	28.9%	0.24	89.28 [85.38-92.69]	0.3008
≥T1b	4	77	65	35.3%	0.20	88.82 [78.82-96.46]	
Location							
Upper third	6	129	113	9.0%	0.36	90.42 [82.96-96.26]	0.3678
Middle third	5	199	173	54.3%	0.07	89.94 [81.35-96.38]	
Lower third	5	143	133	0	0.86	94.99 [89.85-98.67]	
Macroscopic type							
Elevated	3	226	213	86.0%	<0.01	92.26 [78.05-99.75]	0.9924
Flat	3	27	25	40.0%	0.19	96.21 [77.93-100.00]	
Depressed	4	228	208	35.7%	0.20	94.26 [88.37-98.46]	
Histological type							
Differentiated	3	280	252	73.1%	0.02	92.78 [84.87-98.10]	0.6800
Undifferentiated	6	341	303	79.2%	<0.01	91.76 [82.34-98.20]	
*H. pylori* status							
Noneradicated	4	385	345	90.6%	<0.01	85.52 [70.27-96.10]	0.8187
Uninfected	3	61	56	65.4%	0.06	96.78 [75.30-100.00]	
Eradicated	4	204	187	86.7%	<0.01	90.36 [74.04-99.51]	
Endoscopists' experience							
Less experienced	1	31	28	NA	NA	90.32 [76.92-98.70]	0.8521
Experienced	4	631	573	91.0%	<0.01	88.08 [77.38-9.577]	

CI: confidence interval; T1a: mucosal cancer; T1b: submucosal cancer; NA: not available.

**Table 3:** The negative resection rate of the horizontal margin in differentiated-type EGC and undifferentiated-type EGC.

	Number of studies	*n* (lesions examined)	Events (lesions examined)	*I* ^2^	*P*	95% CI (%)	*P* value
Differentiated	2	404	402	64.5%	0.09	99.77 [98.23-100.00]	0.2528
Undifferentiated	5	335	314	89.4%	<0.01	97.40 [89.06-100.00]	

EGC: early gastric cancer; CI: confidence interval.

## Data Availability

No data were used to support this study.

## Supplementary Materials

Supplementary Materials The supplementary file contains the questions of QUADAS, the quality of the included articles, and the forest plot of sensitivity analysis. (Supplementary Materials)
