# Supplementary material for: Diagnostic Ability of Magnifying Narrow-Band Imaging for the Extent of Early Gastric Cancer: A Systematic Review and Meta-Analysis
Source: Gastroenterol Res Pract. 2021 Apr 23;2021:5543556. doi: 10.1155/2021/5543556 (PMC8093039; doi:10.1155/2021/5543556)
Supplement: Supplementary Materials — The supplementary file contains the questions of QUADAS, the quality of the included articles, and the forest plot of sensitivity analysis. (Supplementary Materials) [file 5543556.f1.pdf]

Supplementary data 1 : The questions of the Quality Assessment of Diagnostic Accuracy Studies

The questions of the Quality Assessment of Diagnostic Accuracy Studies (QUADAS) were shown as followed:
Item 1: Was the spectrum of patients representative of the patients who will receive the test in practice?<br>Item 2: Were selection criteria clearly described?<br>Item 3: Is the reference standard likely to correctly classify the target condition?<br>Item 4: Is the time period between reference standard and index test short<br>enough to be reasonably sure that the target condition did not change between the two tests?<br>Item 5: Did the whole sample or a random selection of the sample receive verification by using a reference standard of diagnosis?<br>Item 6: Did patients receive the same reference standard regardless of the index test result?<br>Item 7: Was the reference standard independent of the index test?<br>Item 8: Was the execution of the index test described in sufficient detail to permit replication of the test?<br>Item 9: Was the execution of the reference standard described in suffcient detail to permit its replication?<br>Item 10: Were the index test results interpreted without knowledge of the results of the reference standard?<br>Item 11: Were the reference standard results interpreted without knowledge of the results of the index test?<br>Item 12: Were the same clinical data available when test results were interpreted as would be available when the test is used in practice?<br>Item 13: Were uninterpretable/intermediate test results reported?<br>Item 14: Were withdrawals from the study explained?

Supplementary data 2: Quality of the ten articles included in the meta-analysis using the QUADAS tool

Study	Item 1	Item 2	Item 3	Item 4	Item 5	Item 6	Item 7	Item 8	Item 9	Item 10	Item 11	Item 12	Item 13	Item 14	Score
Yoshimizu et al. [14]	N	Y	Y	Y	Y	Y	Y	Y	Y	Y	U	Y	Y	Y	12
Horii et al. [17]	N	U	Y	Y	Y	Y	Y	Y	Y	Y	Y	Y	Y	Y	12
Horiuchi et al. [16]	N	Y	Y	Y	Y	Y	Y	Y	Y	Y	Y	Y	U	Y	12
Nagahama et al. [15]	N	Y	Y	Y	Y	Y	Y	Y	Y	Y	Y	Y	Y	Y	13
Horiguchi et al. [9]	N	U	Y	Y	Y	Y	Y	Y	Y	Y	Y	Y	Y	U	11
Asada-Hirayama et al. [12]	N	Y	Y	Y	Y	Y	Y	Y	Y	Y	Y	Y	Y	Y	13
Horiuchi et al. [13]	N	Y	Y	Y	Y	Y	Y	Y	Y	Y	U	Y	Y	Y	12
Nonaka et al. [10]	N	U	Y	Y	Y	Y	Y	Y	Y	Y	U	Y	Y	U	10
Okada et al. [11]	N	U	Y	Y	Y	Y	Y	Y	Y	Y	Y	Y	Y	Y	12
Kiyotoki et al. [18]	N	Y	Y	Y	Y	Y	Y	U	Y	Y	Y	U	U	Y	10

Y yes, *N* no, *U* unclear, *QUADAS* Quality Assessment of Diagnostic Accuracy Studies

Supplementary data 3 : Forest plot of sensitive analysis

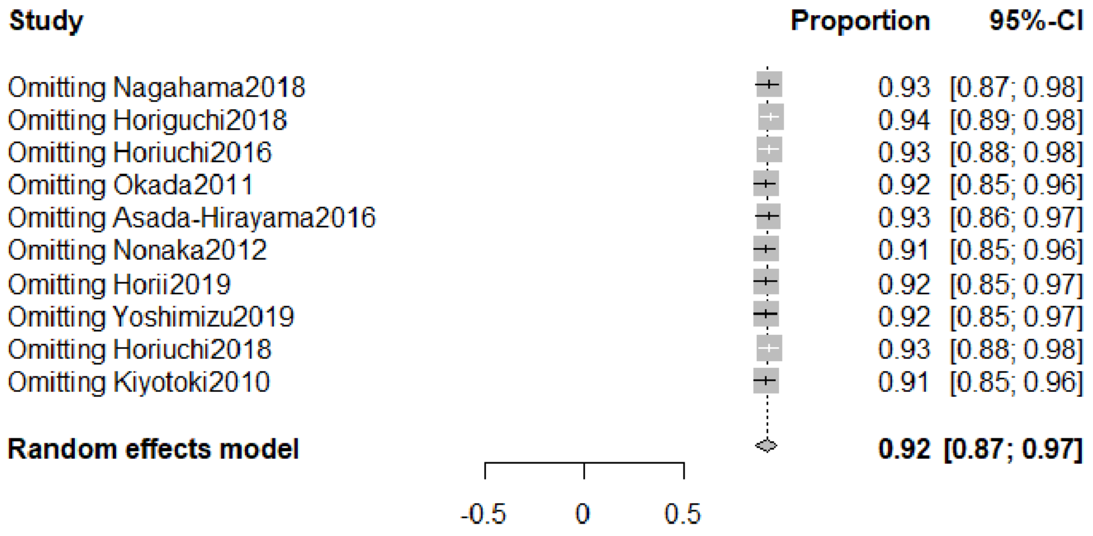

Forest plot showing the result of sensitive analysis for the diagnostic accuracy of ME-NBI for the extent of EGC. *ME-NBI* magnifying endoscopy with narrow-band imaging, *EGC* early gastric cancer
